# Suprachoroidal silicone oil

**DOI:** 10.4103/0974-620X.60025

**Published:** 2010

**Authors:** S. Deka, H. Bhattacharjee

**Affiliations:** Vitreoretina Service, Sri Sankaradeva Nethralaya, Beltola, India

Dear Sir,

Silicone oil (SO), first used by Cibis *et* al.[1] in early 1960s, has an important role in modern vitreoretinal surgery. We report a case of intraoperative suprachoroidal SO and its management.

A 47-year-old man presented with sudden painless loss of vision in his left eye (OS) of 19 days duration. On examination, best corrected visual acuity (BCVA) was counting fingers close to face OS. Fundus examination showed a rhegmetogenous retinal detachment (RD) with a shallow choroidal detachment. The patient underwent pars-plana vitrectomy with SO injection. During SO-air exchange, suprachoroidal SO infusion was noted as a choroidal elevation. SO-air exchange was stopped and location of the infusion cannula checked. Once the tip of the cannula was confirmed to be in the vitreous cavity, SO injection was restarted to completely fill the vitreous cavity with SO. During early postoperative period, the patient′s intra ocular pressure (IOP) was normal and retina was attached. Choroidal elevation due to suprachoroidal SO was seen in fundus periphery [Figure 1a]. The patient underwent SO removal, membrane removal and repeat-SO injection for recurrent RD with proliferative vitreoretinopathy after two months. During SO removal, suprachoroidal SO removal was done by negotiating the superotemporal sclerotomy without entering the vitreous cavity. Ringer lactate solution was infused into the vitreous cavity simultaneously. Subsequently, the patient underwent routine SO removal with epiretinal membrane removal four months after the second surgery. The final BCVA two months after the third surgery was 6/24, N: 10. The fundus picture showed attached retina [Figure 1b].

**Figure 1a:**
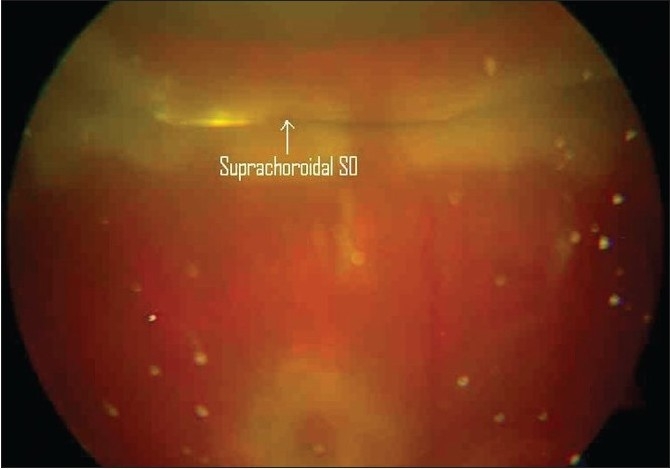
Fundus picture of Suprachoroidal silicone oil (arrow)

**Figure 1b:**
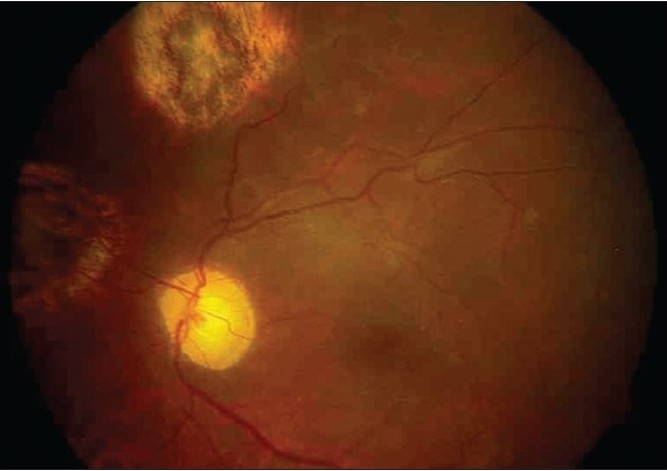
Fundus picture of attached retina after removal of suprachoroidal silicone oil. Chorioretinal scars at retinotomy sites are seen

Under normal circumstances, hydrostatic pressure in the suprachoroidal space is equal to the IOP.[2] We postulate that transient hypotony towards the end of the SO-air exchange caused a local choroidal detachment, allowing the tip of the infusion cannula to enter the suprachoroidal space. An inadvertent pull on the infusion cannula and simultaneous injection of the SO could also have initiated a separation between the choroid and the sclera, allowing egress of silicone oil into the suprachoroidal space. Since retina was seen to be attached at the end of surgery, silicone oil tamponade was considered to be sufficient, and no further intervention was done.

The sequelae of suprachoroidal silicone probably depend on the amount and location of suprachoroidal silicone. A large volume may result in ciliary detachment and hypotony; a more centrally located suprachoroidal SO can affect the vision. However, a small amount of SO away from the macula may be well tolerated as was noted in our case. Although the effects of SO on the retina remain controversial,[3] there are no reported toxic effects of SO on the choroid. A recurrent RD in our patient two months after initial surgery necessitated resurgery. During this procedure, the suprachoroidal SO was removed. Although complete removal was attempted, small emulsified globules were difficult to remove.

The eye remained stable at the six month follow-up examination after suprachoroidal SO removal. Suprachoroidal SO is a rare complication; SO can be removed through a sclerotomy without entering the vitreous cavity during routine SO removal procedure.
